# Detrimental effects of sleep deprivation on the regulatory mechanisms of postural balance: a comprehensive review

**DOI:** 10.3389/fnhum.2023.1146550

**Published:** 2023-04-13

**Authors:** Thierry Paillard

**Affiliations:** Laboratoire Mouvement, Equilibre, Performance et Santé (UPRES EA 4445), Université de Pau et des Pays de l’Adour, E2S, Tarbes, France

**Keywords:** posture, postural control, sleep, acute sleep deprivation, chronic sleep deprivation, fatigue, vision, vestibular system

## Abstract

This review addresses the effects of sleep deprivation on postural balance based on a comprehensive search of articles dealing with this relationship in the electronic databases PubMed, Google Scholar, and ScienceDirect. Evidence suggests that postural balance is sensitive to acute and chronic sleep deprivation for everyone, including young and healthy subjects. Pathologies, aging and the circadian pattern aggravate and/or accentuate the effects of sleep deprivation on postural balance. It turns out that the different systems of information taking, decision making, and motor execution of the postural balance function are negatively affected by sleep deprivation. For example, regarding the information taking system, the sensitivity of visual perception and visuo-spatial performance and the oculomotricity are disrupted and the vestibulo-ocular reflex and the sensory reweighting are altered. Regarding the decision making system, the different brain areas activated for the regulation of postural balance are less active after sleep deprivation and the executive function and perception of verticality are impaired. Regarding the motor execution system, the agonist-antagonist muscle coordination can be modified. However, the different detrimental effects induced for each system of the postural balance function are not yet fully known and deserve further exploration in order to better understand them.

## Introduction

Fatigue can be defined as the degradation of functional capabilities (e.g., motor, cognitive) as a result of excessive physical/physiological and/or mental/psychological workloads (Gandevia, 2001; Van Cutsem et al., 2017). This degradation occurs even if the quantity and quality of sleep enable the restoration of optimal working capacity prior to complete fatiguing workloads/tasks. Alternatively, in the absence of fatiguing physical or mental workload, sleep deprivation alone is likely to degrade functional capabilities (Thun et al., 2015). In fact, sleep deprivation disturbs functional capabilities whether people are physically and/or mentally active or even inactive (sedentary) during the daytime. Sleep deprivation affects anyone in any type of physical or mental activity requiring a greater or lesser investment (degree of involvement) in performance contexts (e.g., artistic, sport, military, or professional) and in the simple/ordinary activities of daily life (Patel et al., 2008; Goel et al., 2013).

Sleep deprivation may cause physiological (e.g., muscle strength or power, energy supply) and neurobehavioral (e.g., attention, information taking, decision making, reaction time, and response accuracy) changes likely to disturb any motor expression, as basic or specialized as it may be, in terms of motor output or skills (Fullagar et al., 2015; Cullen et al., 2019; Kirshner et al., 2022). It would seem logical that motor output and/or skills that strongly mobilize physiological and neurobiological resources (e.g., intense sports or motor activities) are negatively affected when these latter are disturbed by sleep deprivation (Fullagar et al., 2015; Thun et al., 2015). However, it would seem more surprising that basic motor activities requiring few physiological and cognitive resources, such as postural balance, are disturbed by sleep deprivation (Patel et al., 2008). In fact, it turns out that the observed experimental results are opposite to those suggested above. Indeed, sleep deprivation affects submaximal (postural balance or movement control task) but not maximal (peak aerobic or neuromuscular exercise) physical performances (Vaara et al., 2018). Skurvydas et al. (2020) reinforced this idea by specifying that sleep deprivation does not change maximal muscle strength or gross motor performance when movement control precision is not necessary. Sleep deprivation would thus particularly affect submaximal activities that require precision and high control of movements (Thun et al., 2015) such as postural balance (Patel et al., 2008; Kirshner et al., 2022).

Based on these previous considerations, it is logical that it has been suggested that the disturbance of postural balance caused by sleep deprivation may increase the risk of falling in humans (Knechel and Chang, 2022). There would even be a relationship between sleep deprivation (or sleep disturbance/restriction) and falls in adults (Knechel and Chang, 2022). Moreover, sleep deprivation increases the risk of road traffic accidents (Herman et al., 2014; Cai et al., 2021). Overall, sleep deprivation can be considered a public health problem concerning, for example, the worlds of sport and art with the stress induced by competition and shows, frequency and duration of displacements and jet lag; night workers with the chronobiological desynchronization likely to be induced; older people with increased risks of insomnia; people with obstructive sleep apnea syndrome, anxiety and depression, fibromyalgia, and many other pathologies etc., (e.g., Van Reeth and Mennuni, 2002; Waterhouse et al., 2004; Leatherwood and Dragoo, 2013; Umemura et al., 2018; Serrano-Checa et al., 2020).

The above-mentioned disruptive effects of sleep deprivation and the consequences on postural balance are relatively well known. However, the underlying mechanisms are little known and still poorly understood. Knowing that sleep deprivation can be characterized by effects on postural balance, informed explanations of these impairing effects within the regulatory mechanism of postural balance would advance knowledge of the relationship between sleep deprivation and neurobehaviour.

Two systematic reviews have already reported the adverse effects of sleep deprivation on postural balance (Izadi et al., 2022; Umemura et al., 2022) while another systematic review stated a positive relationship between sleep quality and postural balance (Kirshner et al., 2022). These latter authors suggested most physiological and psychological factors are likely to influence this relationship but the mechanistic explanations remain to be precisely described to better understand and prevent the risk of falls, accidents and work-related injuries. Therefore, the present work aims to provide insight into the adverse effects of sleep deprivation on postural balance regulation based on mechanistic explanations.

## Basis of analysis and methodology

### Theoretical support related to postural balance

#### Characterization of postural balance

Posture describes the position of the different segments of the body while balance corresponds to maintaining the body’s center of mass above the base of support in order to avoid a fall (or an imbalance). Hence, postural balance can be defined as the ability to maintain a particular segmental organization without falling (Paillard, 2017a). It can be assessed on the basis of the ability to maintain body balance in ecological and/or challenging postures, i.e., any type of posture, or the ability to minimize continuous body sway in conventional or standard postures, i.e., standing postures (Paillard, 2017a).

Postural balance is classically assessed in bipodal and/or monopodal postures. These assessments are based on kinetic and kinematic measurements (center of foot pressure, center of mass, body segment displacements described in terms of amplitude and/or velocity in the 2–3 dimensions/directions of space -anteroposterior and mediolateral if there are 2) and/or electromyographic measurements. These measurements are taken with eyes open and/or eyes closed, on a stable or instable base, with or without displacement of pedal support (Paillard and Noe, 2015). However, other functional or clinical tests are also used to evaluate postural balance based on success criterion (pass or fail) or on assessment scale (different levels of performance) by carrying out motor tasks in different postures (e.g., lying, sitting, and standing) (Paillard and Noe, 2015). Depending on the nature of the protocols (experimental or clinical), the authors use kinetic and/or kinematic tests (with or without electromyographic measurements) or functional/clinical tests.

#### Regulatory mechanisms of postural balance

Postural balance regulation requires information taking (vestibular, visual, cutaneous, and proprioceptive inputs), processing this information, decision making (central integration and command), and motor execution (activation of antigravity muscles). This regulation is based on neuronal loops that function according to hierarchical and stereotyped patterns and depend on cognitive function and internal body representation or body representation in space (Massion, 1994). Internal body representation is built on body geometry (position and orientation of the segments serve as a reference frame for perception and action with respect to the environment), kinetics (friction between the plantar cutaneous surface and the ground, acceleration of the body), body orientation and vertical perception (with reference to the subjective vertical) cues (Paillard, 2012). Actions require matching multisensory inputs regulating orientation and stabilization of body segments with compensated (*a posteriori*, i.e., during and after postural disturbance) and anticipated (*a priori*, i.e., prior to postural disturbance) postural adjustments (postural responses that are spontaneous and/or included in motor programs) (Paillard, 2019).

From a mechanistic viewpoint, the different cues stemming from vestibular, visual, cutaneous and proprioceptive inputs are integrated at the level of vestibular nuclei (representing the integration center) that ensure vestibulo-ocular, vestibulo-spinal, and vestibulo-cerebellar connexions (Naranjo et al., 2016). The ocular motor activity is based on vestibulo-ocular and visuo-ocular reflexes (Bronstein, 2016). At the spinal level, facilitator and inhibitory neurons regulate different spinal reflexes such as stretch, tendinous, ipsilateral flexion, and contralateral extension reflexes (Bloem et al., 2000). Motor responses need to be selected, triggered, controlled, and coordinated by the basal ganglia (selection of movement), cerebellum (control of movement), and primary motor cortex (triggering of movement) which set up the control center of postural balance (Richard et al., 2017). These motor responses are carried out by antigravity muscles located in the axial and/or proximal part of the head, trunk, thigh, leg, and foot (in particular type I muscle fibers) while their antagonists are inhibited by inhibitory neurons through the reciprocal inhibition reflex (Paillard, 2017b). Axial musculature is connected to the reticulospinal tract (which facilitates anticipatory postural adjustments) and the vestibulospinal tract (which regulates compensatory postural adjustments) as well as part of the corticospinal tract, while distal musculature is under the control of the red nucleus and the rubrospinal tract as well as a part of the corticospinal tract ensuring fine postural motor actions (Paillard, 2017b). Overall, the cortical structures control voluntary postural responses *via* a long loop while the subcortical and spinal structures ensure automatic and reflex postural responses through medium and short loops, respectively, (Jacobs and Horak, 2007; Zaback et al., 2022).

### Characterization of sleep deprivation

Sleep deprivation is the result of insufficient sleep quantity and/or quality required to fully restore physiological and psychological functions (which are individually highly variable but generally require 7–9 h of sleep per day). Most of the time the effects of sleep deprivation (or sleep restriction/disturbance) on postural balance are studied in acute application (short term), i.e., after a full (or partial) night of sleep deprivation with continuous wakefulness. The effects of sleep deprivation in chronic application are also studied over several consecutive days (or even several consecutive weeks or months) and are mostly analyzed in the context of chronic poor sleep quality (e.g., obstructive sleep apnea). In order to focus strictly on the relationship between sleep deprivation and postural balance, any fatiguing physical or mental workload and any stimulating and/or disturbing substance (e.g., caffeine, alcohol) should be avoided during the continuous wakefulness period (to avoid the cumulative or interference effects) and prior testing.

### Information sources on the relationship between postural balance and sleep deprivation

A comprehensive search for articles dealing with the effects of sleep deprivation on postural balance was conducted (at the beginning of 2022) using the keywords “postural balance,” “posture,” “sleep deprivation,” “sleep restriction,” “sleep disorders,” “proprioceptive cues/information,” “visual cues/information,” “vestibular cues/information,” “motor response,” and “executive function” using the electronic databases PubMed, Google Scholar, and ScienceDirect.

## Relationship between postural balance and sleep

Evidence suggests that acute sleep deprivation negatively impacts postural balance on stable or instable base of support, with eyes open and/or eyes closed, or in functional clinical conditions (e.g., Avni et al., 2006; Ma et al., 2009; Siu et al., 2015; Moon et al., 2018; Vaara et al., 2018). Even a partial sleep restriction of 4 h can negatively affect postural balance in young subjects (Souissi et al., 2020). Day-to-day deterioration in sleep quantity and quality (decreased duration, increased fragmentation, increased nocturnal activity) also disturbs postural balance (Montesinos et al., 2018). Conversely, subjects with no alteration in sleep quantity and quality present no change in postural balance (Montesinos et al., 2018). In fact, only sleep pattern variations over consecutive days may affect balance (Montesinos et al., 2018). Montesinos et al. (2018) pointed out that variations in sleep quality cause higher sympathetic activity (e.g., heart rate variability) during the sleep opportunity, which suggests the presence of more wake intervals and/or arousals, and fewer and/or shorter deep sleep intervals. Regression models indicate sleep quality would be a predictor of postural balance (Kowalski et al., 2021). Poor sleep quality would be associated with greater postural sway in the closed eyes and firm surface postural condition than the other conditions - open eyes, foam surface - (Kowalski et al., 2021).

Moreover, a 60 min nap improves postural balance (Ammar et al., 2021). It is already known that napping overcomes the cognitive and physical deterioration induced by sleep deprivation and leads to a reduced level of perceived sleepiness, but it would also produce a better integration of visual and vestibular cues and a better motor output in the postural balance regulation which would be degraded after sleep deprivation (Ammar et al., 2021).

## Main factors influencing the effects of sleep deprivation on postural balance

Pathologies, aging, sex, and circadian patterns influence the effects of sleep deprivation on postural balance. Certain pathologies, such as fibromyalgia and Marfan syndrome, cause somatosensory disorders that negatively affect postural balance (Akkaya et al., 2013; Micarelli et al., 2019).

Aging also modifies the effects of sleep deprivation on postural balance. Sleep deprivation would have greater destabilizing effects on postural balance in older than in younger subjects and could thus increase the risk of falls in aged subjects (Robillard et al., 2011). One can deduce that sleep deprivation would accentuate the adverse effects of advancing age on postural balance (Olpinska-Lischka et al., 2021). Olpinska-Lischka et al. (2021) stated that women would be better able to cope with the effects of sleep deprivation than men, although this deserves to be confirmed.

Moreover, it is generally known that circadian patterns impact postural balance (e.g., Morad et al., 2007). Postural balance fluctuates according to a rhythm which is close to that of body temperature and/or vigilance (Bougard et al., 2011). The different circadian phases of rectal temperature could directly lead to variations in the functioning of the muscles used for postural balance (Nakano et al., 2001). Logically, postural balance should also vary throughout the day in sleep deprivation (Bougard et al., 2011). According to Bougard et al. (2011), the most pronounced periods are in the middle of the day (10:00 a.m. and 2:00 p.m.). This would explain why the initial deterioration of postural balance after 24 h of sleep deprivation was not followed by a further deterioration in postural performance after 36 h of sleep deprivation (Patel et al., 2008). However, for internal body representation, Martin et al. (2018) reported that sleep deprivation caused no diural variation related to subjective visual vertical and postural balance. Sleep deprivation worsened the mean estimation of the subjective visual vertical and postural balance and flattened the diurnal fluctuation of the subjective visual vertical, leading to the disappearance of the diurnal rhythm (Martin et al., 2018). Finally, the chronotype of the subject (e.g., morning chronotype performs better in morning tests than evening chronotype) can also constitute another factor influencing the effects of sleep deprivation on postural balance (Umemura et al., 2022).

## Chronic poor sleep quality and postural balance

Chronic poor sleep quality impairs postural balance similarly to total sleep deprivation-one night without sleep- (Furtado et al., 2016; Tanwar et al., 2021). Poor sleep quality not only affects postural balance, but is also associated with high levels of anxiety and depression (Serrano-Checa et al., 2020). Among the reasons for chronic poor sleep quality, obstructive sleep apnea is a major problem. Periodic collapse of the upper airway during sleep in patients with obstructive sleep apnea causes chronic intermittent hypoxia and sleep interruption (Gokmen et al., 2021). Obstructive sleep apnea and insomnia are associated with an increased risk of falls (Gokmen et al., 2021; Knechel and Chang, 2022). It is not clear whether daytime sleepiness, self-rated sleep quality, snoring or napping are associated with falls, as some, but not all, and studies show an association between these variables (Knechel and Chang, 2022). When severe obstructive sleep apnea-impaired static postural balance is compared to mild to moderate (non-severe) obstructive sleep apnea, there was no difference between them in terms of dynamic postural balance and the risk of fall (Gokmen et al., 2021). Nocturnal hypoxia may cause deterioration in static postural balance as the apnea-hypopnea index increases and nocturnal blood oxygen saturation level decreases (Gokmen et al., 2021). Sleep-disordered breathing severity, especially the mean nocturnal blood oxygen saturation level, is associated with impaired daytime postural balance (Degache et al., 2016). Degache et al. (2016) suggested that hypoxemia would degrade postural balance at least partially by altering the muscle spindle reactivity so essential to postural regulation. Gokmen et al. (2021) reported that hypoxia and sleep interruption are thought to provoke cardiovascular and cerebrovascular diseases and brain damage with oxidative stress, sympathetic activation, and systemic and vascular inflammation. All this damage and alterations related to the systems of information taking, the decision making and the motor execution of the postural and balance function are likely to degrade postural balance.

## Effects of sleep deprivation on the different systems of the postural balance function

### Information taking system

#### Visual function

Evidence suggests that sleep deprivation has less effect on postural balance with eyes open than with eyes closed both in anteroposterior and lateral direction (Patel et al., 2008). The anteroposterior direction is nevertheless more affected than the mediolateral direction (Umemura et al., 2019). Nocturnal changes in postural balance are more sensitive to sleepiness when the eyes are closed than when they are open because of the postural compensatory effects of visual information (Liu et al., 2001). The disruptive effects of sleep deprivation are more pronounced in older subjects than in young subjects under altered visual condition (Robillard et al., 2011) as well as in men than in women (Olpinska-Lischka et al., 2021).

Although visual information limits the postural disturbance, this additional information seems not to be sufficient to compensate fully for the alteration of postural balance caused by sleep deprivation (Gomez et al., 2008; Patel et al., 2008). Eyes closed subjects with worse sleep quality also had worse postural balance (Furtado et al., 2016). Lack of vision deeply impairs postural balance in subjects with chronic sleep insufficiency. Moreover, Cheng et al. (2018) showed that postural balance began to deteriorate after 16 h of sleep deprivation with eyes open and after 28 h of sleep deprivation with eyes closed, using a foam platform (i.e., under altered proprioceptive condition). This suggests that the effects of sleep deprivation evolve over time and that compensatory mechanisms related to sensory reweighting are modified differently depending on whether they are based on visual cues or not.

**FIGURE 1:**
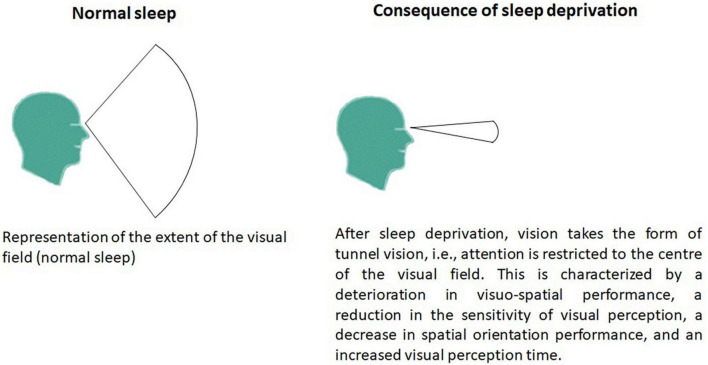
Representation of the visual field without and with (consequence) sleep deprivation.

Of course, the contribution of visual information strongly impacts postural balance in the context of sleep deprivation but the visual field is also important. Schlesinger et al. (1998) observed that sleep deprivation disturbed postural balance only during a task requiring the intermittent inhibition of a reaction but not with a simple reaction time task and in the absence of a concurrent information processing task. Sleep deprivation deteriorates the useful visual field according to a general interference phenomenon (Rogé et al., 2003). This deterioration of the useful visual field is progressive (Figure 1). This can take the form of tunnel vision (i.e., attention is restricted to the center of the visual field) when the sleep deprivation is low (a few hours) but progresses to general interference (involving the whole of the useful visual field) when sleep deprivation is higher (Rogé et al., 2003). Moreover, in the context of a tunnel-vision task (experimental task), sleep deprivation resulted in an overall slowing of reaction times and increased errors of omission for both peripheral and foveal stimuli (Jackson et al., 2008). Visual perception time was prolonged after sleep deprivation in young healthy adults (Batuk et al., 2020). Sleep deprivation was associated with electrophysiological evidence of altered higher cognitive processes (attention and visual information processing), whereas early processing of visual information did not appear to be affected (Jackson et al., 2008; Batuk et al., 2020). In fact, the effects following sleep deprivation are related to changes in later cognitive rather than early sensory processing (Jackson et al., 2008). However, these tunnel-vision effects are not exacerbated by sleep deprivation. Jackson et al. (2008) suggested that sleep loss has a general effect on attention allocation to visual stimuli on a global level, with no specific effects on the location of the stimuli.

Overall, sleep deprivation leads to a deterioration in visuo-spatial performance, a reduction in the sensitivity of visual perception, a decrease in spatial orientation performance and spatial data storage (Besnard et al., 2018; Vargas et al., 2020). Acute sleep loss not only reduced visual task performance and impaired visuospatial perception but also disrupted selective attention, probably due to a reduction in the downward bias of information processing in the sensory cortex (Dyakova et al., 2019). All these disturbances related to the visual function negatively affect postural balance, especially under dynamic conditions (i.e., unstable support, moving head, and moving visual scene). Hence, tasks requiring substantial attention to cognitive and motor demands are degraded more than tasks that are automatic and were learned-retention of learning was not degraded by sleep deprivation- (Kaplan et al., 2017). The more complex the postural task, the more postural balance is degraded. Sleep deprived individuals became less stable and less acurate in relating visual information to motor actions (Aguiar and Barela, 2014). Individuals have limited capability to select the most relevant visual cues in order to produce appropriate motor actions, demonstrated by their higher variability in body oscillation (Aguiar and Barela, 2014). In addition, they place less importance or weight on visual cues and more importance on cues from other sensory channels, resulting in lower gain values for visual information after sleep deprivation condition (Aguiar and Barela, 2015).

#### Ocular motor function

Saccadic eye movements improve postural balance even in sleep-deprived subjects but are still not sufficient to avoid postural balance deterioration due to sleep deprivation (Vargas et al., 2020). Vargas et al. (2020) specified that sleep-deprived subjects reduced postural sway magnitude when using horizontal saccades compared to when only fixating a stationary target. Horizontal saccades are not sufficient to overcome the deleterious effects of sleep deprivation (Vargas et al., 2020). This limited deterioration of postural balance following sleep deprivation could stem from the synergistic relationship between the postural function and the visual function (Bonnet and Baudry, 2016). Bonnet and Baudry (2016) inferred that the relationship between these two functions would be congruent rather than competitive. Based on these considerations, Vargas et al. (2020) proposed that there is no dual relation between these two functions even in sleep-deprived subjects. It can thus be suggested that although sleep deprivation negatively affects postural balance, it does not alter the saccadic eye movements that facilitate it. Moreover, sleep deprivation deteriorated smooth pursuit gain, smooth pursuit accuracy and saccade velocity (Fransson et al., 2008). However, a more detailed analysis shows an interesting result which illustrates that the ratio between saccade velocity and saccade amplitude was decreased by sleep deprivation. In fact, only smooth pursuit gain deteriorated whereas there were signs of improvement in smooth pursuit accuracy measurements (Fransson et al., 2008). Knowing that ocular motor activity also results indirectly from the vestibular system *via* the vestibular nuclei (integration center), it seem relevant to tackle the role of the vestibular function as part of sleep deprivation.

#### Vestibulo-ocular function

**FIGURE 2:**
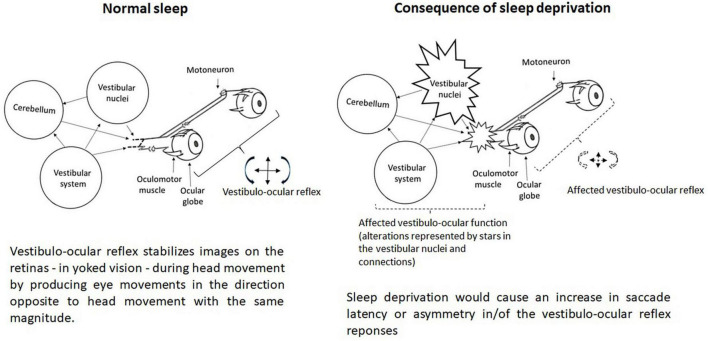
Representation of the vestibulo-ocular function without and with (consequence) sleep deprivation.

Sleep deprivation has a real impact on the vestibular system and visual perception time in young adults (Batuk et al., 2020). It would specifically affect the vestibulo-ocular reflex (Quarck et al., 2006). Quarck et al. (2006) highlighted that sleep deprivation would affect only the latency of saccade and the smooth pursuit gains, not the accuracy of the saccade. Saccade velocity is affected in any saccade kind after one night of sleep deprivation (Zils et al., 2005). Collins (1988) had previously observed that, depending on the velocity of the phase (slow or fast), the durations of nystagmus are likely to differ significantly. Later, Quarck et al. (2006) observed, on the basis of an experiment in which the rotation was 60°s^–1^ velocity, that sleep deprivation induced an increase in the vestibulo-ocular reflex. In a second experiment these authors reported that with a sinusoidal rotation (0.2 Hz ± 25°s^–1^) sleep deprivation induced no significant modification in vestibulo-ocular reflex gain. Based on the work of Quarck et al. (2006), sleep-deprived subjects showed an increase in eye velocity in the slow phase of the vestibulo-ocular reflex triggered by a velocity step and not by a sinusoidal rotation. These results are not shared by Lin and Young (2014) as they found that the effect of short duration sleep deprivation (12 h, after night duty at the emergency service) on the vestibulo-ocular reflex, or after a normal sleep, showed no difference between the two sleep conditions i.e., latencies and amplitude of ocular vestibular-evoked myogenic potential. However, Lin and Young (2014) observed that the mean asymmetry ratio after sleep deprivation was larger than after normal sleep. Hence, sleep deprivation would accentuate the vestibulo-ocular reflex asymmetry (Lin and Young, 2014). Obviously, the effects of sleep deprivation on vestibular function remain relatively complex (Figure 2).

The vestibular nuclei are connected *via* the inter-geniculate lateral region with the supra-chiasmatic hypothalamic nucleus, i.e., the basis of biological rhythmicity which is modulated by different synchronizing inputs, especially light, as well as synchronizing social influences (Besnard et al., 2018). Besnard et al. (2018) pointed out that the vestibular sensory organs, particularly the otolithic receptors, also play a synchronizing role in the biological rhythms as there would be a reciprocal neuroanatomical pathway between the vestibular nuclei and the orexinergic neurons. Hence, Besnard et al. (2018) postulated that vestibular inputs, by modulating the sleep-wake state, would influence postural balance since there is a direct reciprocal connection between the vestibular nuclei and a number of vestibular-related cortical areas that extend from the posterior parietal cortex to the frontal regions.

#### Sensory reweighting

As previously mentioned, sensory reweighting in the absence of visual cues is altered by sleep deprivation (Cheng et al., 2018). However, the contribution of proprioceptive information could be less influential on postural balance than that of visual information in the context of sleep deprivation since sleep quality was not associated with postural balance on foam support (Kowalski et al., 2021). Sensory manipulations can generate other considerations. Although sleep deprivation affects unperturbed standing, the most prominent effects were observed when sleep-deprived subjects were exposed to proprioceptive vibratory stimulation (Gomez et al., 2008). This would indicate a decreased ability to adapt to balance perturbation following sleep deprivation. Gomez et al. (2008) suggested that this may be related to the level of attention of sleep deprived subjects. Calf vibration provides stimuli that engenders a illusion of movement. Under normal conditions (without sleep deprivation), the subject’s attentional state is sufficiently high (efficient) to reweight the different sensory inputs and grant more importance to the most reliable sensory receptors. The ability to prioritize sensory inputs would be degraded during calf vibration in sleep-deprived individuals, as evidenced by greater variations in segmental body movements (Gomez et al., 2008). Since the integration of different sensory information requires attention and sleep deprivation lessens attention, Gomez et al. (2008) and Patel et al. (2008) inferred that sensory integration would lower or be in appropriated in the sleep deprivation context. Thus, the ability to choose the most relevant motor response (segmental adjustment) to maintain/ensure postural balance would be degraded under the sleep deprivation condition.

### Decision making system

#### Brain function

There are negative correlations between postural sway and the electroencephalographic alpha activity (Liu et al., 2001). Hence, it is logical that the brain areas activated for the regulation of postural balance are less active after sleep deprivation since postural sway increase (Fabbri et al., 2006). On the one hand, sleep deprivation may induce disturbances in the brain sensory integration areas and, on the other hand, the areas of the cerebral cortex that regulate aspects of attention, alertness and cognitive ability, such as thalamus and regions within the prefrontal and anterior cingulate cortices, undergo deactivation periods following 24 h of sleep deprivation (e.g., Furtado et al., 2016; Kowalski et al., 2021). As the sleep deprivation becomes longer, the brain areas such as the thalamus and the prefrontal cortex are less activated probably due to induced brain tissue hypometabolism (Orzeł-Gryglewska, 2010). Sleep deprivation would affect the metabolism of the thalamus, cerebellum and basal ganglia, affecting sensory integration and motor coordination and therefore postural balance (Umemura et al., 2022). Hence, a reduced activation of the thalamus and the prefrontal cortex may cause input integration disturbances related to a lack attentional resources and reduced supervision function (Fabbri et al., 2006; Martin et al., 2018). Moreover, activation of the extrastriate cortex and lateral occipital sections of the interparietal sulcus would be reduced following sleep deprivation (Batuk et al., 2020). The number of functional cortical circuits in processing visual information would then be decreased which would affect many important visual functions (e.g., the processing of rapidly changing visual information, visual selectivity, suppression of distracting visual information, and visual short-term memory) following sleep deprivation (Batuk et al., 2020). Moreover, the hippocampus is altered by sleep deprivation as well as hippocampus-cortical functional connectivity (Zhao et al., 2019a). Sleep-deprived subjects thus exhibit lower hippocampal functional connectivity (Zhao et al., 2019a). According to the time of day, supported by the chronobiological rhythm, compensatory mechanisms may take place between the various altered cerebral resources and may entail an increase in vigilance which would reduce postural balance disturbances (Bougard et al., 2011).

#### Executive function

Evidence suggests that, with sleep deprivation, impaired executive function is likely to affect postural balance, particularly under dynamic and challenging postural conditions, since compensated postural adjustments following a postural disturbance must occur as quickly as possible to avoid a fall or body imbalance (Paillard, 2017a). It is already well known that sleep deprivation impairs reaction time (Jackson et al., 2008; Daviaux et al., 2014; Fullagar et al., 2015; Vaara et al., 2018; Zhao et al., 2019b). While it is possible that sleep deprivation does not degrade simple reaction time, it negatively affects cognitive function or performance (Skurvydas et al., 2020). For examples, time pressure increases error rate and vigilance is deteriorated (Bonnet and Arand, 2003; Fullagar et al., 2015). Perceptual output for action capabilities is altered during prolonged wakefulness because sleep deprivation impairs the central executive processes, with adverse effects on attention and response inhibition (Daviaux et al., 2014). However, Sagaspe et al. (2003) reported that 36 h of prolonged wakefulness did not appear to significantly affect inhibition processes in a short executive task. In fact, changes in motor performance due to sleep deprivation depend not only on the type of task, but also on the sleep deprivation protocol. Sleep deprivation worsened attention and inhibition control during the Go/No-Go task but did not change attention and inhibitory control during the Stroop task (Skurvydas et al., 2021). Overall, sleep deprivation affects a wide range of cognitive domains (including attention, working memory, abstraction, and decision making) and results in decreases in both the encoding of new information and memory consolidation (Goel et al., 2013). Hence, sleep deprivation would not be conducive to the learning of new postural balance skills, which is essential for the improvement of postural balance (aimed at improving motor performance or preventing falls in athletes, workers or older subjects, respectively), since it relies more on the sum of specifically learned postural skills than on a general development of postural balance that can be improved independently of the balance tasks that have been learned in the past (Kümmel et al., 2016). Specific balance training improves particular postural balance skills (Paillard, 2017b).

Since sleep deprivation particularly affects submaximal motor tasks that require high control of movements, such as postural balance mentioned above, it would be logical that the cognitive and sensory-motor impairment associated with sleep deprivation (Thun et al., 2015) contributes to impaired postural balance. In fact, sleep deprivation has deleterious effects on the sensory-motor ability necessary to consider one’s action capabilities to ensure safe and efficient movement control (Daviaux et al., 2014). Daviaux et al. (2014) argued that the cognitive processing of external (i.e., environmental cues) and internal (i.e., the subject’s physical state) inputs that specify the estimated consequences of motor action could explain the inability to successfully update the perception of action capabilities after sleep deprivation. In addition, sleep deprivation would have a negative effect on the subjective visual vertical, which is important in the process of postural balance regulation (Martin et al., 2018).

### Motor execution system

#### Spinal function

Since the contribution of the myotatic loops are fundamental in postural balance regulation, it is worth considerating the effects of sleep deprivation on the spinal reflexes. The effects of sleep deprivation on the stretch reflex and tendinous reflex are relatively unknown. The effects of sleep deprivation were assessed with respect to the spinal excitability by Gonçalves et al. (2021) who reported that H-reflex (sensitive to changes in presynaptic inhibition and/or motoneuron excitability) and V-wave (sensitive to modification in supraspinal input to the motoneuron pool) peak-to-peak normalized amplitude did not change after sleep deprivation, revealing that the descending neural drive and/or modulation in Ia afferent input remained unaffected under sleep deprivation condition. Nevertheless, Daviaux et al. (2014) previously showed that the Hmax reflex was lowered after one night of sleep deprivation, although no change was observed regarding maximal voluntary contraction (quadriceps femoris). Based on these contradictory results and the fact that sleep deprivation affects movement control, it can be assumed that the motor system of the postural balance function could be negatively affected.

#### Neuromuscular function

Different neuromuscular parameters related to the motor function or motor output likely to impact postural balance were evaluated under sleep deprivation conditions. Some parameters are durably sustained and measured under rest conditions while other parameters are only briefly sustained and measured over short periods under maximal/peak or dynamic conditions.

Among the parameters durably sustained, muscle tone greatly influences postural balance, so that enhanced muscle tone minimizes postural segmental movements and improves postural balance (Paillard, 2017b). Orzeł-Gryglewska (2010) reported that sleep deprivation intensifies muscle tone. Hence, this intensification would at least partially compensate for the disturbance of postural balance resulting from the lack of sleep. However, Sá Gomes e Farias et al. (2022) observed that sleep deprivation was associated with an increase in muscle sympathetic nervous activity. Due to its excitatory effects, cardiac, vascular, and respiratory muscles are more strongly activated, i.e., modulation of peripheral vascular resistance, venous return, heart rate, contractility, and cardiac output as well as respiratory variables (Sá Gomes e Farias et al., 2022). All these factors amplify the internal reaction forces that constitute internal mechanical stimuli and contribute to small continuous segmental displacements and thus disturb postural balance (Conforto et al., 2011). Hence, the factors which increase muscle tone are likely to be outweighed by the factors which augmente muscle sympathetic nervous activity.

Other parameters directly related to motor output, such as maximum voluntary contraction (i.e., maximal muscle strength), rate of force development (i.e., the speed at which the contractile proteins of the muscle can develop force which reflects the capability to develop as much as forces it possible in a short period of time) and central activation ratio (i.e., the ability to fully and voluntarily contract a muscle group), were assessed as part of sleep deprivation. Skurvydas et al. (2021) observed that maximum voluntary contraction of knee extensor muscles was reduced but not rate force development and central activation ratio. Daviaux et al. (2014), however, reported no changes in actual performance (maximum voluntary contraction of quadriceps femoris and height jump). These authors postulated that the organism would activate protective mechanisms by altering the inhibition process under sleep deprivation conditions to maintain physical performance in maximal tasks. Overall, the absolute or relative (time-dependent) level of force production does not decrease or decreases so little after sleep deprivation that it would not contribute to disturbing postural balance. However, it is worth considering the effects of sleep deprivation on the muscle coordination (e.g., agonist/antagonist muscle activity), which could subtly or substantially impact movement control tasks such as postural balance.

**FIGURE 3:**
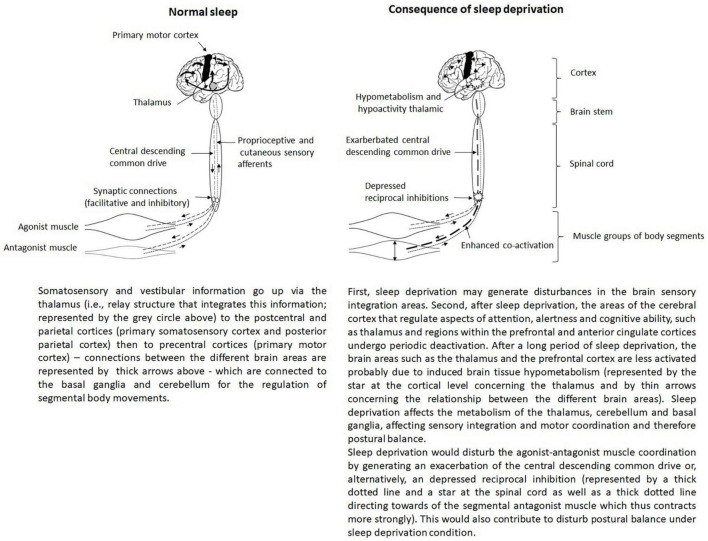
Representation of the regulation of postural balance tasks involving the central integration of sensory inputs and motor responses.

Gonçalves et al. (2021) showed that the antagonist/agonist level of co-contraction or co-activation increased during maximal voluntary contraction following sleep deprivation. This change of the antagonist/agonist co-activation would mean that the muscle coordination was affected after sleep deprivation. The enhanced co-activation at maximal force levels may be mediated by an exacerbation of the central descending common drive or, alternatively, depressed reciprocal inhibition (Gonçalves et al., 2021). Despite the obvious differences between both control mechanisms, in either case the central nervous system would end up generating a greater level of excitability of the antagonist motoneurons and thus increased co-activation (Figure 3). Sleep deprivation would affect movement control by exacerbating the magnitude of antagonist/agonist co-activation during intense muscle contractions. This phenomenon presents advantages and disadvantages for postural balance (Paillard, 2017b). Indeed, two opposing forces exerted on a joint can enhance the stiffness of the joint and thus its stability, which improves postural balance by generating a compensatory mechanism. Conversely, augmented co-activation would interlock body segments and impede the ability to finely control them. This would lead more or less forced changes in segmental postural strategies/mobilizations and thus to a disturbing postural balance (Paillard, 2017b). Overall, since postural balance is impaired under sleep deprivation conditions, the different compensatory mechanisms would probably not compensate for the disturbing effects and would suggest that sleep deprivation would have a negative effect on the motor system of postural balance function.

Sleep deprivation causes additional disadvantages in older subjects compared to young subjects. Robillard et al. (2011) postulated that age-related muscle atrophy would degrade postural muscles’ efficiency to a greater extent in older subjects than in younger subjects in a sleep deprived context compared to a normal/ordinary context. This is likely to result in maladaptive anticipatory and/or compensatory postural adjustments in older subjects due to low motor output/efficiency exacerbated under sleep deprivation conditions, which alters their postural balance and thus further increases their risk of falling especially when they are initially frail and already have very low muscle output (Paillard, 2017a).

## Conclusion

On the basis of all the data presented above, the postural balance function and its different systems of information taking, decision making and motor execution turn out to be sensitive to a state of sleep deprivation and is of interest in any behavioral study carried out in relation to sleep quantity and/or quality. Sleep deprivation disrupts the regulatory mechanisms of postural balance. Its disruptive effects occur at the level of each system of the postural balance function. However, the different disruptive effects induced for each system are not yet fully known and deserve further exploration in order to better understand them.

## Author contributions

The author confirms being the sole contributor of this work and has approved it for publication.

## References

[B1] Aguiar S. A. Barela J. A. (2014). Sleep deprivation affects sensorimotor coupling in postural control of young adults. *Neurosci. Lett.* 574 47–52. 10.1016/j.neulet.2014.05.028 24858135

[B2] Aguiar S. A. Barela J. A. (2015). Adaptation of sensorimotor coupling in postural control is impaired by sleep deprivation. *PLoS One* 10:e0122340. 10.1371/journal.pone.0122340 25799560 PMC4370556

[B3] Akkaya N. Akkaya S. Atalay N. S. Acar M. Catalbas N. Sahin F. (2013). Assessment of the relationship between postural stability and sleep quality in patients with fibromyalgia. *Clin. Rheumatol.* 32 325–331. 10.1007/s10067-012-2117-y 23179001

[B4] Ammar A. Boukhris O. Hsouna H. Dhia I. B. Trabelsi K. Gujar T. A. (2021). The effect of a daytime 60-min nap opportunity on postural control in highly active individuals. *Biol. Sport* 38 683–691. 10.5114/biolsport.2021.104067 34937979 PMC8670816

[B5] Avni N. Avni I. Barenboim E. Azaria B. Zadok D. Kohen-Raz R. (2006). Brief posturographic test as an indicator of fatigue. *Psychiatry Clin. Neurosci.* 60 340–346. 10.1111/j.1440-1819.2006.01511.x 16732751

[B6] Batuk I. T. Batuk M. O. Aksoy S. (2020). Evaluation of the postural balance and visual perception in young adults with acute sleep deprivation. *J. Vestib. Res.* 30 383–391. 10.3233/VES-200778 33285660

[B7] Besnard S. Tighilet B. Chabbert C. Hitier M. Toulouse J. Le Gall A. (2018). The balance of sleep: Role of the vestibular sensory system. *Sleep Med. Rev.* 42 220–228. 10.1016/j.smrv.2018.09.001 30293919

[B8] Bloem B. R. Allum J. H. Carpenter M. G. Honegger F. (2000). Is lower leg proprioception essential for triggering human automatic postural responses? *Exp. Brain Res.* 130 375–391. 10.1007/s002219900259 10706436

[B9] Bonnet C. T. Baudry S. (2016). Active vision task and postural control in healthy, young adults: Synergy and probably not duality. *Gait Posture* 48 57–63. 10.1016/j.gaitpost.2016.04.016 27477709

[B10] Bonnet M. H. Arand D. L. (2003). Clinical effects of sleep fragmentation versus sleep deprivation. *Sleep Med. Rev.* 7 297–310. 10.1053/smrv.2001.0245 14505597

[B11] Bougard C. Lepelley M. C. Davenne D. (2011). The influences of time-of-day and sleep deprivation on postural control. *Exp. Brain Res.* 209 109–115. 10.1007/s00221-010-2524-8 21188358

[B12] Bronstein A. M. (2016). Multisensory integration in balance control. *Handb. Clin. Neurol.* 137 57–66. 10.1016/B978-0-444-63437-5.00004-2 27638062

[B13] Cai A. W. T. Manousakis J. E. Singh B. Kuo J. Jeppe K. J. Francis-Pester E. (2021). On-road driving impairment following sleep deprivation differs according to age. *Sci. Rep.* 11:21561. 10.1038/s41598-021-99133-y 34732793 PMC8566466

[B14] Cheng S. Ma J. Sun J. Wang J. Xiao X. Wang Y. (2018). Differences in sensory reweighting due to loss of visual and proprioceptive cues in postural stability support among sleep-deprived cadet pilots. *Gait Posture* 63 97–103. 10.1016/j.gaitpost.2018.04.037 29727778

[B15] Collins W. (1988). Some effects of sleep loss on vestibular responses. *Aviat. Space Environ. Med.* 59 523–529. 3260486

[B16] Conforto S. Schmid M. Camomilla V. D’Alessio T. Cappozzo A. (2011). Hemodynamics as a possible internal mechanical disturbance to balance. *Gait Posture* 14 28–35. 10.1016/S0966-6362(01)00112-6 11378422

[B17] Cullen T. Thomas G. Wadley A. J. Myers T. (2019). The effects of a single night of complete and partial sleep deprivation on physical and cognitive performance: A Bayesian analysis. *J. Sports Sci.* 37 2726–2734. 10.1080/02640414.2019.1662539 31608829

[B18] Daviaux Y. Mignardot J. B. Cornu C. Deschamps T. (2014). Effects of total sleep deprivation on the perception of action capabilities. *Exp. Brain Res.* 232 2243–2253. 10.1007/s00221-014-3915-z 24691757

[B19] Degache F. Goy Y. Vat S. Haba Rubio J. Contal O. Heinzer R. (2016). Sleep-disordered breathing and daytime postural stability. *Thorax* 71 543–548. 10.1136/thoraxjnl-2015-207490 26892395

[B20] Dyakova O. Rångtell F. H. Tan X. Nordström K. Benedict C. (2019). Acute sleep loss induces signs of visual discomfort in young men. *J. Sleep Res.* 28:e12837. 10.1111/jsr.12837 30815934 PMC6900002

[B21] Fabbri M. Martoni M. Esposito M. J. Brighetti G. Natale V. (2006). Postural control after a night without sleep. *Neuropsychologia* 44 2520–2525. 10.1016/j.neuropsychologia.2006.03.033 16690088

[B22] Fransson P. A. Patel M. Magnusson M. Berg S. Almbladh P. Gomez S. (2008). Effects of 24-hour and 36-hour sleep deprivation on smooth pursuit and saccadic eye movements. *J. Vest. Res.* 18 209–222. 10.3233/VES-2008-18404 19208965

[B23] Fullagar H. H. Skorski S. Duffield R. Hammes D. Coutts A. J. Meyer T. (2015). Sleep and athletic performance: The effects of sleep loss on exercise performance, and physiological and cognitive responses to exercise. *Sports Med.* 45 161–186. 10.1007/s40279-014-0260-0 25315456

[B24] Furtado F. da Silva B. Gonçalves B. Lopes Laguardia Abranches I. Flávia Abrantes A. Forner-Cordero A. (2016). Chronic low quality sleep impairs postural control in healthy adults. *PLoS One* 11:e0163310. 10.1371/journal.pone.0163310 27732604 PMC5061348

[B25] Gandevia S. C. (2001). Spinal and supraspinal factors in human muscle fatigue. *Physiol. Rev.* 81 1725–1789. 10.1152/physrev.2001.81.4.1725 11581501

[B26] Goel N. Basner M. Rao H. Dinges D. F. (2013). Circadian rhythms, sleep deprivation, and human performance. *Progr. Mol. Biol. Transl. Sci.* 119 155–190. 10.1016/B978-0-12-396971-2.00007-5 23899598 PMC3963479

[B27] Gokmen G. Y. Gurses H. N. Zeren M. Ozyilmaz S. Kansu A. Akkoyunlu M. E. (2021). Postural stability and fall risk in patients with obstructive sleep apnea: A cross-sectional study. *Sleep Breath* 25 1961–1967. 10.1007/s11325-021-02322-2 33608798

[B28] Gomez S. Patel M. Berg S. Magnusson M. Johansson R. Fransson P. A. (2008). Effects of proprioceptive vibratory stimulation on body movement at 24 and 36h of sleep deprivation. *Clin. Neurophysiol.* 119 617–625. 10.1016/j.clinph.2007.10.058 18164660

[B29] Gonçalves A. D. Teodosio C. Pezarat-Correia P. Vila-Chã C. Mendonca G. V. (2021). Effects of acute sleep deprivation on H reflex and V wave. *J. Sleep Res.* 30:e13118. 10.1111/jsr.13118 32567138

[B30] Herman J. Kafoa B. Wainiqolo I. Robinson E. McCaig E. Connor J. (2014). Driver sleepiness and risk of motor vehicle crash injuries: A population-based case control study in Fiji (TRIP 12). *Injury* 45 586–591. 10.1016/j.injury.2013.06.007 23830198 PMC3969304

[B31] Izadi M. Thomas E. Thomas A. C. Bellafiore M. (2022). The effect of time-of-day and sleep deprivation on postural control: A systematic review. *Gait Posture* 97 94–103. 10.1016/j.gaitpost.2022.07.245 35917703

[B32] Jackson M. L. Croft R. J. Owens K. Pierce R. J. Kennedy G. A. Crewther D. (2008). The effect of acute sleep deprivation on visual evoked potentials in professional drivers. *Sleep* 31 1261–1269. 18788651 PMC2542966

[B33] Jacobs J. V. Horak F. B. (2007). Cortical control of postural responses. *J. Neural Transm.* 114 1339–1348. 10.1007/s00702-007-0657-0 17393068 PMC4382099

[B34] Kaplan J. Ventura J. Bakshi A. Pierobon A. Lackner J. R. DiZio P. (2017). The influence of sleep deprivation and oscillating motion on sleepiness, motion sickness, and cognitive and motor performance. *Auton. Neurosci*. 202 86–96. 10.1016/j.autneu.2016.08.019 27641791

[B35] Kirshner D. Spiegelhalder K. Shahar R. T. Shochat T. Agmon M. (2022). The association between objective measurements of sleep quality and postural control in adults: A systematic review. *Sleep Med. Rev.* 63:101633. 10.1016/j.smrv.2022.101633 35504085

[B36] Knechel N. A. Chang P. S. (2022). The relationships between sleep disturbance and falls: A systematic review. *J. Sleep Res.* 15:e13580. 10.1111/jsr.13580 35288982

[B37] Kowalski K. L. Boolani A. Christie A. D. (2021). State and trait fatigue and energy predictors of postural control and gait. *Motor Control* 25 519–536. 10.1123/mc.2020-0106 34117130

[B38] Kümmel J. Kramer A. Giboin L. S. Gruber M. (2016). Specificity of balance training in healthy individuals: A systematic review and meta-analysis. *Sports Med.* 46 1261–1271. 10.1007/s40279-016-0515-z 26993132

[B39] Leatherwood W. E. Dragoo J. L. (2013). Effect of airline travel on performance: A review of the literature. *Br. J. Sports Med*. 47 561–567. 10.1136/bjsports-2012-091449 23143931

[B40] Lin B. Y. Young H. Y. (2014). Effect of short-duration sleep deprivation on the vestibulo-ocular reflex system evaluated by ocular vestibular-evoked myogenic potential test. *Acta Otolaryngol.* 134 698–703. 10.3109/00016489.2014.895039 24834933

[B41] Liu Y. Higuchi S. Motohashi Y. (2001). Changes in postural sway during a period of sustained wakefulness in male adults. *Occup. Med.* 51 490–495. 10.1093/occmed/51.8.490 11741080

[B42] Ma J. Yao Y. J. Ma R. M. Li J. Q. Wang T. Li X. J. (2009). Effects of sleep deprivation on human postural control, subjective fatigue assessment and psychomotor performance. *J. Int. Med. Res.* 37 1311–1320. 10.1177/147323000903700506 19930836

[B43] Martin T. Gauthier A. Ying Z. Benguigui N. Moussay S. Bulla J. (2018). Effect of sleep deprivation on diurnal variation of vertical perception and postural control. *J. Appl. Physiol*. 125 167–174. 10.1152/japplphysiol.00595.2017 29543136

[B44] Massion J. (1994). Postural control system. *Curr. Opin. Neurobiol.* 4 877–887. 10.1016/0959-4388(94)90137-6 7888772

[B45] Micarelli A. Viziano A. Lanzillotta A. Giorgino F. M. Pisano C. Ruvolo G. (2019). Postural control abnormalities related to sleep deprivation in patients with Marfan Syndrome. *J. Vestib. Res*. 29 261–269. 10.3233/VES-190684 31707379

[B46] Montesinos L. Castaldo R. Cappuccio F. P. Pecchia L. (2018). Day-to-day variations in sleep quality affect standing balance in healthy adults. *Sci. Rep.* 8:17504. 10.1038/s41598-018-36053-4 30504839 PMC6269497

[B47] Moon Y. I. Yoon S. Y. Jeong Y. J. Cho T. H. (2018). Sleep disturbances negatively affect balance and gait function in post-stroke patients. *NeuroRehabilitation* 43 211–218. 10.3233/NRE-172351 30040752

[B48] Morad Y. Azaria B. Avni I. Barkana Y. Zadok D. Kohen-Raz R. (2007). Posturography as an indicator of fatigue due to sleep deprivation. *Aviat. Space Environ. Med.* 78 859–863. 17891895

[B49] Nakano T. Araki K. Michimori A. Inbe H. Hagiwara H. Koyama E. (2001). Nineteen-hour variation of postural sway, alertness and rectal temperature during sleep deprivation. *Psychiatry Clin. Neurosci.* 55 277–278. 10.1046/j.1440-1819.2001.00858.x 11422874

[B50] Naranjo E. N. Cleworth T. W. Allum J. H. Inglis J. T. Lea J. Westerberg B. D. (2016). Vestibulo-spinal and vestibulo-ocular reflexes are modulated when standing with increased postural threat. *J. Neurophysiol*. 115 833–842. 10.1152/jn.00626.2015 26631147

[B51] Olpinska-Lischka M. Kujawa K. Maciaszek J. (2021). Differences in the effect of sleep deprivation on the postural stability among men and women. *Int. J. Environ. Res. Pub. Health* 18:3796. 10.3390/ijerph18073796 33916500 PMC8038654

[B52] Orzeł-Gryglewska J. (2010). Consequences of sleep deprivation. *Int. J. Occup. Med. Environ. Health* 23 95–114. 10.2478/v10001-010-0004-9 20442067

[B53] Paillard T. (2012). Effects of general and local fatigue on postural control: A review. *Neurosci. Biobehav. Rev.* 36 162–176. 10.1016/j.neubiorev.2011.05.009 21645543

[B54] Paillard T. (2017a). Relationship between muscle function, muscle typology and postural performance according to different postural conditions in young and older adults. *Front. Physiol.* 8:585. 10.3389/fphys.2017.00585 28861000 PMC5559497

[B55] Paillard T. (2017b). Plasticity of the postural function to sport and/or motor experience. *Neurosci. Biobehav. Rev.* 72 129–152. 10.1016/j.neubiorev.2016.11.015 27894829

[B56] Paillard T. (2019). Relationship between sport expertise and postural skills. *Front. Psychol.* 10:1428. 10.3389/fpsyg.2019.01428 31293483 PMC6603331

[B57] Paillard T. Noe F. (2015). Techniques and methods for testing the postural function in healthy and pathological subjects. *Biomed Res. Int.* 2015:891390. 10.1155/2015/891390 26640800 PMC4659957

[B58] Patel M. Gomez S. Berg S. Almbladh P. Lindblad J. Petersen H. (2008). Effects of 24-h and 36-h sleep deprivation on human postural control and adaptation. *Exp. Brain Res.* 185 165–173. 10.1007/s00221-007-1143-5 17932662

[B59] Quarck G. Ventre J. Etard O. Denise P. (2006). Total sleep deprivation can increase vestibulo-ocular responses. *J. Sleep Res.* 15 369–375. 10.1111/j.1365-2869.2006.00550.x 17118093

[B60] Richard A. Van Hamme A. Drevelle X. Golmard J. L. Meunier S. Welter M. L. (2017). Contribution of the supplementary motor area and the cerebellum to the anticipatory postural adjustments and execution phases of human gait initiation. *Neuroscience* 358 181–189. 10.1016/j.neuroscience.2017.06.047 28673716

[B61] Robillard R. Prince F. Filipini D. Carrier J. (2011). Aging worsens the effects of sleep deprivation on postural control. *PLoS One* 6:e28731. 10.1371/journal.pone.0028731 22163330 PMC3233602

[B62] Rogé J. Pébayle T. El Hannachi S. Muzet A. (2003). Effect of sleep deprivation and driving duration on the useful visual field in younger and older subjects during simulator driving. *Vision Res.* 43 1465–1472. 10.1016/S0042-6989(03)00143-3 12767314

[B63] Sá Gomes e Farias A. V. Peixoto de Lima Cavalcanti M. Alcântara de Passos M. Del Vechio Koike B. (2022). The association between sleep deprivation and arterial pressure variations: A systematic literature review. *Sleep Med.* 4:100042. 10.1016/j.sleepx.2022.100042 35169694 PMC8829775

[B64] Sagaspe P. Charles A. Taillard J. Bioulac B. Philip P. (2003). Inhibition and working memory: Effect of acute sleep deprivation on a random letter generation task. *Can. J. Exp. Psychol.* 57 265–273. 14710864

[B65] Schlesinger A. Redfern M. S. Dahl R. E. Jennings J. R. (1998). Postural control, attention and sleep deprivation. *Neuroreport* 9 49–52. 10.1097/00001756-199801050-00010 9592046

[B66] Serrano-Checa R. Hita-Contreras F. Jiménez-García J. D. Achalandabaso-Ochoa A. Aibar-Almazán A. Martínez-Amat A. (2020). Sleep quality, anxiety, and depression are associated with fall risk factors in older women. *Int. J. Environ. Res. Pub. Health* 17:4043. 10.3390/ijerph17114043 32517112 PMC7312056

[B67] Siu K. C. Huang C. K. Beacom M. Bista S. Rautiainen R. (2015). The association of sleep loss and balance stability in farmers. *J. Agromed.* 20 327–331. 10.1080/1059924X.2015.1042615 26237723

[B68] Skurvydas A. Kazlauskaite D. Zlibinaite L. Cekanauskaite A. Valanciene D. Karanauskiene D. (2021). Effects of two nights of sleep deprivation on executive function and central and peripheral fatigue during maximal voluntary contraction lasting 60s. *Physiol. Behav.* 229:113226. 10.1016/j.physbeh.2020.113226 33122092

[B69] Skurvydas A. Zlibinaite L. Solianik R. Brazaitis M. Valanciene D. Baranauskiene N. (2020). One night of sleep deprivation impairs executive function but does not affect psychomotor or motor performance. *Biol. Sport* 37 7–14. 10.5114/biolsport.2020.89936 32205905 PMC7075226

[B70] Souissi N. Zouita A. Abedelmalek S. Trabelsi K. Cain C. Clark T. (2020). Partial sleep restriction impairs static postural control in elite judo athletes. *Biol. Rhythm Res.* 53 653–664. 10.1080/09291016.2020.1843254

[B71] Tanwar T. Veqar Z. Ghrouz A. K. Spence D. W. Pandi-Perumal S. R. (2021). Is poor sleep quality associated with a deterioration in postural control? *Sleep Sci*. 14 207–213. 10.5935/1984-0063.20200061 35186198 PMC8848529

[B72] Thun E. Bjorvatn B. Flo E. Harris A. Pallesen S. (2015). Sleep, circadian rhythms, and athletic performance. *Sleep Med. Rev*. 23 1–9. 10.1016/j.smrv.2014.11.003 25645125

[B73] Umemura G. S. Furtado F. Dos Santos F. C. Gonçalves B. D. S. B. Forner-Cordero A. (2022). Is balance control affected by sleep deprivation? A systematic review of the impact of sleep on the control of balance. *Front. Neurosci.* 16:779086. 10.3389/fnins.2022.779086 35651634 PMC9150847

[B74] Umemura G. S. Pinho J. P. da Silva Brandão Gonçalves B. Furtado F. Forner-Cordero A. (2018). Social jetlag impairs balance control. *Sci. Rep.* 8:9406. 10.1038/s41598-018-27730-5 29925863 PMC6010412

[B75] Umemura G. S. Pinho J. P. Santos J. P. F. C. Forner-Cordero A. (2019). Assessment of postural control after sleep deprivation with a low-cost portable force plate. *Annu. Int. Conf. IEEE Eng. Med. Biol. Soc.* 2019 2316–2319. 10.1109/EMBC.2019.8857561 31946363

[B76] Vaara J. P. Oksanen H. Kyröläinen H. Virmavirta M. Koski H. Finni T. (2018). 60-Hour sleep deprivation affects submaximal but not maximal physical performance. *Front. Physiol.* 16:1437. 10.3389/fphys.2018.01437 30386253 PMC6198717

[B77] Van Cutsem J. Marcora S. De Pauw K. Bailey S. Meeusen R. Roelands B. (2017). The effects of mental fatigue on physical performance: A systematic review. *Sports Med.* 47 1569–1588. 10.1007/s40279-016-0672-0 28044281

[B78] Van Reeth O. Mennuni G. (2002). Fatigue and sleep: The point of view of the chronobiologist. *Rev. Méd. Bruxelles* 23 A288–A293. 12422449

[B79] Vargas I. E. P. Bicalho L. E. Rodrigues S. T. Barela J. A. (2020). Saccadic eye movements attenuate postural sway but less in sleep-deprived young adults. *Front. Sports Active Living* 12:97. 10.3389/fspor.2020.00097 33345087 PMC7739768

[B80] Waterhouse J. Reilly T. Edwards B. (2004). The stress of travel. *J. Sports Sci.* 22 946–965. 10.1080/02640410400000264 15768727

[B81] Zaback M. Adkin A. L. Chua R. Inglis J. T. Carpenter M. G. (2022). Facilitation and habituation of cortical and subcortical control of standing balance following repeated exposure to a height-related postural threat. *Neuroscience* 487 8–25. 10.1016/j.neuroscience.2022.01.012 35085706

[B82] Zhao R. Zhang X. Zhu Y. Fei N. Sun J. Liu P. (2019a). Disrupted resting-state functional connectivity in hippocampal subregions after sleep deprivation. *Neuroscience* 398 37–54. 10.1016/j.neuroscience.2018.11.049 30529694

[B83] Zhao R. Zhang X. Fei N. Zhu Y. Sun J. Liu P. (2019b). Decreased cortical and subcortical response to inhibition control after sleep deprivation. *Brain Imag. Behav.* 13 638–650. 10.1007/s11682-018-9868-2 29748772

[B84] Zils E. Sprenger A. Heide W. Born J. Gais S. (2005). Differential effects of sleep deprivation on saccadic eye movements. *Sleep* 28 1109–1115. 10.1093/sleep/28.9.1109 16268380

